# Pancreatoduodenectomy in a patient with celiac axis stenosis and a replaced common hepatic artery: A case report

**DOI:** 10.1016/j.ijscr.2022.107088

**Published:** 2022-04-18

**Authors:** Takashi Komatsubara, Koji Fujimoto, Yuma Tanigawa, Eisei Mitsuoka, Masayuki Isii

**Affiliations:** Department of Surgery, Shinko Hospital, 1-4-47 Wakihama-cho, Chuo-ku, Kobe City, Hyogo 651-0072, Japan

**Keywords:** PD, pancreatoduodenectomy, GDA, gastroduodenal artery, rCHA, replaced common hepatic artery, SMA, superior mesenteric artery, CT, computed tomography, MAL, median arcuate ligament, ICG, indocyanine green, Celiac axis stenosis, Indocyanine green fluorescence imaging, Pancreatoduodenectomy, Replaced common hepatic artery, Case report

## Abstract

**Introduction:**

Evaluation of anatomical variations is important in pancreatoduodenectomy (PD) because an arterial anomaly is a risk factor for morbidity. Herein, we report a rare case of PD for lower bile duct cancer in which celiac axis stenosis and a replaced common hepatic artery (rCHA) coexisted.

**Presentation of case:**

An 84-year-old woman presented with epigastric pain. She was diagnosed with a lower bile duct cancer and underwent PD. Preoperative computed tomography showed celiac axis stenosis, and the deformed celiac artery had a “hooked appearance,” suggesting compression by the median arcuate ligament (MAL). The rCHA branched from the superior mesenteric artery. The gastroduodenal artery (GDA) diverged from the rCHA, forming a developed arterial arcade of the pancreatic head. There was an oncological safety margin between the rCHA and common bile duct; however, a part of the collateral artery was close to the common bile duct. Therefore, we planned to preserve the rCHA and resect the GDA to form collateral circulation. The MAL was divided and we evaluated the blood flow of the left upper abdominal organs using indocyanine green fluorescence imaging with a near-infrared camera system. We considered that perfusion of organs was preserved, and PD was performed without vessel reconstruction. No ischemic complication occurred in the postoperative course.

**Discussion:**

The coexistence of these arterial anomalies made the procedure of PD more complicated.

**Conclusion:**

Precise preoperative diagnosis of arterial anomalies is necessary to avoid postoperative complications that may be induced by intraoperative arterial injury and organ ischemia.

## Highlights

• Median arcuate ligament and a replaced common hepatic artery are rare anomalies.
• A case report of Pancreatoduodenectomy in which these arterial anomalies coexisted
• Arterial injury or insufficient blood flow may induce ischemic complications.
• Precise preoperative diagnosis of arterial anomalies is necessary.

## 1 Introduction

Evaluation of anatomical variations around the pancreas is important in pancreatoduodenectomy (PD) because an arterial anomaly is a risk factor for morbidity [1]. Previous studies have reported celiac axis stenosis on angiography in 12.5–49% of cases [2], [3]. It is usually asymptomatic because of collateral circulation such as the arterial arcade of the pancreatic head. However, dividing these important collaterals via the gastroduodenal artery (GDA) during PD can lead to ischemic complications in the upper abdominal organs [1], [4]. On the other hand, anatomical variations of the hepatic artery system are occasionally observed in 25–40% of cases; nonetheless, a replaced common hepatic artery (rCHA) is rare, with an incidence ranging from 1.5% to 3% [5], [6], [7]. Such arterial variations may be problematic in PD because of the route of the replaced artery near the resection margin, particularly the superior mesenteric artery (SMA) margin. Intraoperative damage to the rCHA may lead to refractory bile leakage from bile duct jejunal anastomosis, liver abscesses [8], [9]. Herein, we report a rare case of PD for lower bile duct cancer in which celiac axis stenosis and rCHA coexisted.

This case report has been written according to the SCARE 2020 criteria [10].

## 2 Presentation of case

**Fig. 1:**
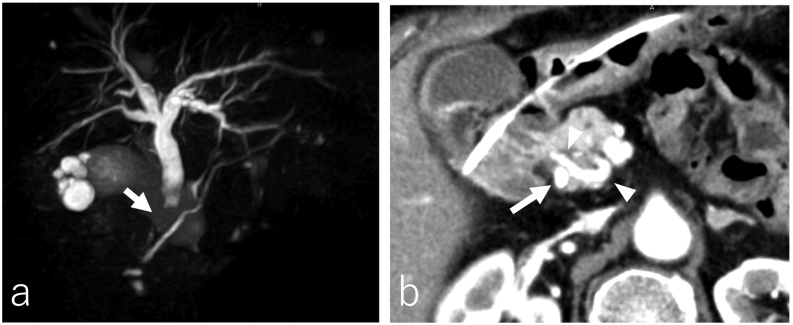
Preoperative imaging findings. (a) Magnetic resonance cholangiopancreatography shows lower bile duct obstruction (arrow) and dilation of the upper bile duct. (b) CT scan after placement of the ENBD tube shows the collateral artery (arrowhead) around the pancreatic head runs in contact with the bile duct in which the ENBD tube was placed (arrow). CT: computed tomography, ENBD: endoscopic nasobiliary drainage.

**Fig. 2:**
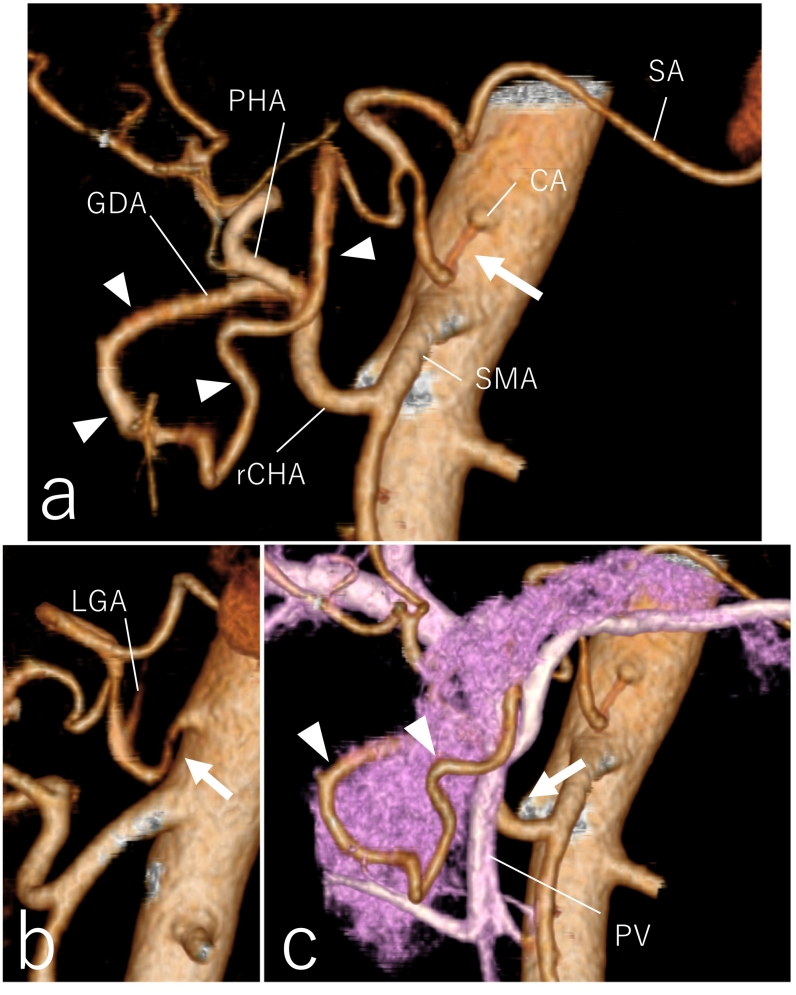
Preoperative CT angiography findings. (a) Front view. Stenosis of the origin of the CA is observed (arrow). The rCHA arises from the SMA and the GDA branches from the rCHA. The collateral pathway of the pancreatic head is developed (arrowhead). (b) Lateral view. Focal stenosis in the proximal celiac axis is described as having a “hooked appearance” (arrow). (c) Three-dimensional CT shows the rCHA passing behind the PV (allow), and the collateral artery runs around the head of the pancreas (arrowhead). CT: computed tomography; CA: celiac artery, rCHA: replaced common hepatic artery, SMA: superior mesenteric artery, GDA: gastroduodenal artery, SA: splenic artery, PHA: proper hepatic artery, LGA: left gastric artery, PV: portal vein.

An 84-year-old woman with a 1-week history of epigastric pain presented to our district general hospital. She had a medical history of hyperlipidemia. Physical examination revealed no fever or jaundice. Laboratory findings indicated a slightly elevated white blood cell count and liver dysfunction. Magnetic resonance cholangiopancreatography showed obstruction of the lower bile duct and dilation of the upper bile duct (Fig. 1(a)). Obstruction due to bile duct cancer was suspected; hence, emergency endoscopic retrograde cholangiopancreatography was performed. An endoscopic nasobiliary drainage tube was placed, and the results of bile duct biopsy indicated adenocarcinoma in the lower bile duct. Contrast-enhanced computed tomography (CT) showed wall thickening of the lower bile duct; however, there was no suspicion of distant metastasis or lymph node metastasis. Stenosis of the origin of the celiac artery was observed, and the deformed celiac artery had a “hooked appearance,” suggesting compression by the median arcuate ligament (MAL) (Fig. 2(a, b)). The common hepatic artery directly branched from the SMA. The rCHA passed through the dorsal side of the portal vein and pancreatic head and branched into the left and right hepatic arteries within the hepatoduodenal mesentery. The GDA diverged from the rCHA, forming a developed arterial arcade of the pancreatic head, which was the collateral pathway between the SMA and celiac artery systems (Fig. 2(a, c)).

**Fig. 3:**
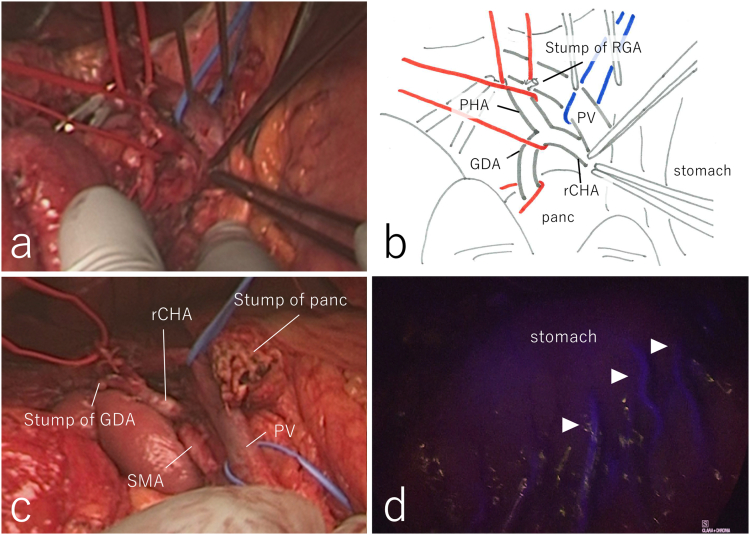
Intraoperative pictures. (a) The GDA branches from the rCHA, which runs behind the PV. (b) Schema of (a). (c) After removal of the duodenum and head of the pancreas. The rCHA arising from the SMA is preserved, and the GDA, which forms collateral circulation, is resected. (d) Indocyanine green fluorescence imaging reveals that the blood supply of stomach is preserved (arrowhead). GDA, gastroduodenal artery, rCHA: replaced common hepatic artery, PV: portal vein, PHA: proper hepatic artery, RGA: right gastric artery, panc: pancreas, SMA: superior mesenteric artery. (For interpretation of the references to colour in this figure legend, the reader is referred to the web version of this article.)

We performed PD with her consent after her liver function was improved by bile duct drainage. Intraoperative exploration ruled out latent peritoneal or liver metastasis and showed arterial anomalies similar to those indicated by preoperative findings. There was an oncological safety margin between the rCHA and common bile duct; however, a part of the collateral artery was close to the common bile duct (Fig. 1(b)). Therefore, we planned to preserve the rCHA and resect the GDA to form collateral circulation (Fig. 3(a–c)). The collateral artery passed the pancreas posteriorly, and joined the celiac artery (Fig. 2(c)). The collateral artery was resected at the root of the celiac artery. Pulsations of the celiac and splenic arteries were attenuated by dissection of the collateral circulation. As the MAL was suspected to be the cause of celiac axis stenosis, division of the MAL was performed. To evaluate blood flow of the left upper abdominal organs, including the stomach, remnant of the pancreas, and spleen, indocyanine green (ICG) fluorescence imaging with a near-infrared camera system was performed. A bolus injection of 5 mg of ICG (Diagnogreen; Dai-Ichi Pharm, Tokyo, Japan) was delivered from a peripheral vein, and blood perfusion was confirmed (Fig. 3(d)). Therefore, PD was performed without vessel reconstruction. The duration of surgery was 493 min, blood loss was 600 mL, and blood transfusion was unnecessary. Pathological findings indicated moderately to poorly differentiated adenocarcinoma of the lower bile duct (T2N0M0, stage IIA, according to the UICC classification).

**Fig. 4:**
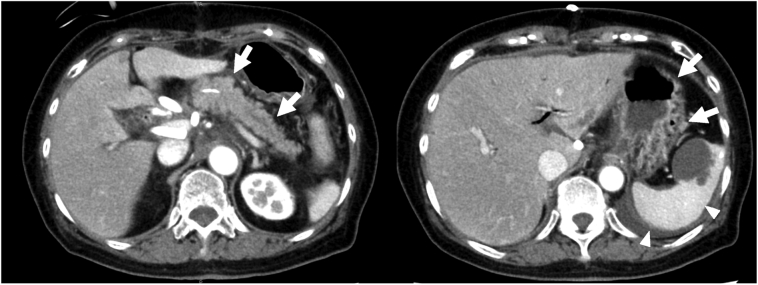
Contrast-enhanced computerized tomography at 1 week after the operation. The contrast effect of the remnant of pancreas, stomach (allow) and spleen (allow head) were preserved.

She developed a postoperative pancreatic fistula (grade B, as defined by the International Study Group on Pancreatic Fistula), which improved with antibiotic administration. Contrast-enhanced CT performed at 1 postoperative week showed that the contrast effect of the stomach, spleen, and remnant pancreas was preserved and no ischemic change was detected (Fig. 4). The patient was discharged on postoperative day 28, and no signs of recurrent cancer were identified at 3 years postoperatively.

## 3 Discussion

PD is an important surgical treatment for tumors located in the head of pancreas, lower bile duct, and duodenum, although morbidity and mortality remain high. Insufficient understanding of anomalies in the celiac artery and SMA systems can lead to intra- and postoperative complications. This is a very rare case in which celiac axis stenosis and a rCHA were recognized on preoperative CT angiography, where a careful intraoperative assessment was required because the coexistence of these anomalies made the procedure of PD more complicated.

It is reported that preoperative CT before PD showed stenosis in 11% of cases [4]. Generally, ischemic symptoms with celiac axis stenosis rarely become a clinical problem, because collateral circulation via the arterial arcade of the pancreatic head develops between the SMA and celiac artery systems. However, dividing these important collaterals during PD can lead to ischemia in the liver, stomach, spleen, and remnant of the pancreas, thereby increasing the risk of complications such as hepatic abscess, bile leakage, residual pancreatic necrosis, pancreatic fistula, and anastomotic leakage at the site of gastrointestinal anastomosis and splenic abscess [11]. Celiac axis compression by the MAL is the most common cause of celiac axis stenosis (55–88%), followed by arteriosclerosis (11–12%), and these two causes account for the majority of cases [4], [12]. The CT angiography finding of characteristic focal narrowing of the proximal celiac axis noted as a “hooked appearance” can help distinguish celiac axis stenosis by the MAL from other causes of celiac axis stenosis, such as atherosclerotic disease [13], [14]. Interventions for celiac axis stenosis have been reported, including division of the MAL, vessel reconstruction, and preservation of collateral circulation [4], [11], [15]. If celiac axis stenosis is due to MAL compression, division of the MAL is considered the primary procedure for surgical treatment. If the arterial flow cannot be resolved because of severe stenosis, revascularization of the celiac artery or preservation of the collateral circulation of the pancreatic head should be considered [16].

During PD in a patient with a replaced hepatic artery which originates from the SMA, the conservative approach is most frequently used unless direct invasion of cancer occurs, because of the risk of thromboembolism and postoperative bleeding caused by a pancreatic fistula [1], [8]. Particularly, in cases with rCHA, the severity of hepatic ischemia following thromboembolism in the reconstructed vessels is increased and may become fatal as the entire liver can become ischemic. Moreover, if celiac axis stenosis coexists with the rCHA, as in our case, the arteries forming an important collateral circulation, such as the GDA and inferior pancreatic duodenal artery, may dilate as much as the hepatic artery. It is difficult to recognize arteries that must be preserved; therefore, more attention should be paid to patient anatomy.

Through an extensive search of PubMed, we identified only one case report concerning PD in a patient with celiac axis stenosis and a rCHA. PD with preservation of the rCHA and collateral circulation of the pancreatic head was performed for intraductal papillary mucinous neoplasia in the pancreatic head [17]. In our case, the rCHA ran through the dorsal side of the portal vein and pancreatic head, and no invasion of the bile duct cancer was observed, but the collateral artery ran close to the common bile duct, as visible in preoperative CT. Consequently, we selected to preserve the rCHA and considered the need for resection of the GDA to form collateral circulation for oncologic cure. After the MAL was divided for celiac axis stenosis, we evaluated the blood flow of the organs by ICG fluorescence imaging to determine the necessity for vessel reconstruction. Recent reports have suggested the use of ICG fluorescence imaging for evaluating the perfusion of various organs, such as in cases of ischemic gastropathy which is a major complication after distal pancreatectomy with celiac axis resection or distal pancreatectomy with distal gastrectomy [18], [19]. In our case, we confirmed that the blood flow of the organs was preserved by ICG fluorescence imaging, which could help us to omit unnecessary vessel reconstruction. Although ICG fluorescence imaging can be easily performed and is noninvasive and valuable, there is concern that ICG fluorescence imaging largely depends on the subjectivity of the operator, and the intensity of fluorescence emitted on the screen is not quantified. The criteria for determining ischemia have not yet been established, and further consideration of cases evaluating blood flow by ICG fluorescence imaging is necessary.

## 4 Conclusion

A precise preoperative diagnosis of arterial anomalies is required to avoid postoperative complications that may be induced by intraoperative arterial injury and organ ischemia.

## Sources of funding

This research did not receive any specific grant from funding agencies in the public, commercial, or not-for-profit sectors.

## Ethical approval

This study was approved by the ethics committee of Shinko Hospital (no. 2126).

## Consent

Written informed consent was obtained from the patient for publication of this case report and accompanying images.

## Author contribution

TK and KF contributed to study conception and design. All authors contributed to data acquisition and analysis. YT and EM were involved in patient treatment. TK and KF were the major contributors to manuscript writing. All authors read and approved the final manuscript.

## Registration of research studies

Not applicable.

## Guarantor

Takashi Komatsubara, corresponding author of this article.

## Provenance and peer review

Not commissioned, externally peer-reviewed.

## Declaration of competing interest

None.
